# Molecular Landscape and Association With Crohn Disease of Poorly Cohesive Carcinomas of the Nonampullary Small Bowel

**DOI:** 10.1093/ajcp/aqac161

**Published:** 2023-02-22

**Authors:** Gianluca Tedaldi, Camilla Guerini, Davide Angeli, Daniela Furlan, Laura Libera, Marco Vincenzo Lenti, Federica Grillo, Matteo Fassan, Enrico Solcia, Fausto Sessa, Marco Paulli, Antonio Di Sabatino, Paola Ulivi, Alessandro Vanoli

**Affiliations:** Biosciences Laboratory, IRCCS Istituto Romagnolo per lo Studio dei Tumori (IRST) “Dino Amadori,” Meldola, Italy; Department of Molecular Medicine, Unit of Anatomic Pathology, University of Pavia, Pavia, Italy; Unit of Biostatistics and Clinical Trials, IRCCS Istituto Romagnolo per lo Studio dei Tumori (IRST) “Dino Amadori,” Meldola, Italy; Pathology Unit, Department of Medicine and Surgery, University of Insubria, Varese, Italy; Pathology Unit, Department of Medicine and Surgery, University of Insubria, Varese, Italy; Department of Internal Medicine, Istituto di Ricovero e Cura a Carattere Scientifico (IRCCS) Fondazione Policlinico San Matteo, University of Pavia, Pavia, Italy; Department of Laboratory Services, IRCCS Ospedale Policlinico San Martino, Genova, Italy; Department of Surgical and Diagnostic Sciences (DISC), Pathology Unit, University of Genova, Genova, Italy; Surgical Pathology and Cytopathology Unit, Department of Medicine-DIMED, University of Padova, Padua, Italy; Veneto Institute of Oncology (I.O.V. IRCCS), Padua, Italy; Department of Molecular Medicine, Unit of Anatomic Pathology, University of Pavia, Pavia, Italy; Pathology Unit, Department of Medicine and Surgery, University of Insubria, Varese, Italy; Department of Molecular Medicine, Unit of Anatomic Pathology, University of Pavia, Pavia, Italy; Unit of Anatomic Pathology, Fondazione IRCCS Policlinico San Matteo, Pavia, Italy; Department of Internal Medicine, Istituto di Ricovero e Cura a Carattere Scientifico (IRCCS) Fondazione Policlinico San Matteo, University of Pavia, Pavia, Italy; Biosciences Laboratory, IRCCS Istituto Romagnolo per lo Studio dei Tumori (IRST) “Dino Amadori,” Meldola, Italy; Department of Molecular Medicine, Unit of Anatomic Pathology, University of Pavia, Pavia, Italy; Unit of Anatomic Pathology, Fondazione IRCCS Policlinico San Matteo, Pavia, Italy

**Keywords:** Crohn disease, Goblet cell adenocarcinoma, Immune-mediated disorders, *RHOA*, Signet ring cell carcinoma

## Abstract

**Objectives:**

Poorly cohesive carcinomas (PCCs) are neoplasms defined by a predominantly dyshesive growth pattern with single cell or cord-like stromal infiltration. The ­distinctive clinicopathologic and prognostic features of small bowel PCCs (SB-PCCs) in comparison with conventional-type small intestinal adenocarcinomas have only recently been characterized. However, as SB-PCCs’ genetic profile is still unknown, we aimed to analyze the molecular landscape of SB-PCCs.

**Methods:**

A next-generation sequencing analysis through Trusight Oncology 500 on a series of 15 nonampullary SB-PCCs was performed.

**Results:**

The most frequently found gene alterations were *TP53* (53%) and *RHOA* (13%) mutations and *KRAS* amplification (13%), whereas *KRAS*, *BRAF*, and *PIK3CA* mutations were not identified. Most SB-PCCs (80%) were associated with Crohn disease, including both *RHOA*-mutated SB-PCCs, which featured a non-SRC-type histology, and showed a peculiar appendiceal-type, low-grade goblet cell adenocarcinoma (GCA)–like component. Rarely, SB-PCCs showed high microsatellite instability, mutations in *IDH1* and *ERBB*2 genes, or *FGFR2* amplification (one case each), which are established or promising therapeutic targets in such aggressive cancers.

**Conclusions:**

SB-PCCs may harbor *RHOA* mutations, which are reminiscent of the diffuse subtype of gastric cancers or appendiceal GCAs, while *KRAS* and *PIK3CA* mutations, commonly involved in colorectal and small bowel adenocarcinomas, are not typical of such cancers.

**KEY POINTS**

- The present study analyzes for the first time the molecular features of poorly cohesive carcinoma of the small bowel (SB-PCCs).
- The most frequent gene alterations in SB-PCCs are *TP53* (53%) and *RHOA* (13%) mutations and *KRAS* amplification (13%).
- *KRAS* and *PIK3CA* point mutations, commonly involved in colorectal and small bowel adenocarcinomas, are lacking in such cancers.

## INTRODUCTION

Despite the extensive surface area of the small intestine, primary nonampullary small bowel adenocarcinomas (SBAs) are rare malignancies, accounting for 31% to 40% of small bowel cancers and about 3% of total incident gastrointestinal tract cancers.^1^ The incidence of SBAs has increased in recent decades in the United States and Europe.^2^ Predisposing conditions for SBAs encompass both immune-mediated disorders, such as celiac disease and Crohn disease, and hereditary syndromes, like Lynch syndrome and familial adenomatous polyposis.

Even though most SBAs show a conventional, glandular-type histology, other histologic subtypes have been described.^3^ In ­particular, SBAs featuring a predominantly dyshesive spread of the neoplastic cells, with single cell or cord-like infiltration, are now designated as poorly cohesive carcinomas (PCCs) in the World Health Organization 2019 Classification of Tumors of the Digestive System, and “poorly cohesive cell carcinoma with or without signet ring cells” has been included as a distinct histologic type in the 2021 College of American Pathologists protocol guidelines.^3,4^ A recent study from our group proved that, when compared with conventional-type glandular SBAs (ie, SBAs not otherwise specified [SBAs-NOS]), small bowel PCCs (SB-PCCs) are characterized by unique clinicopathologic features, such as a younger age at diagnosis, a more frequent association with Crohn disease as a predisposing condition, a higher rate of perineural and lymphovascular invasion, and a worse prognosis.^5^

Although the treatment of SBAs has historically been based on strategies elaborated for colorectal carcinomas (CRCs), patients with SBA have a worse prognosis at all stages, and specific therapeutic options are lacking. Recent studies revealed that SBAs harbor different molecular features from CRCs, including a higher incidence of mismatch repair deficiency (MMRd)/microsatellite instability (MSI) high and *CDKN2A* mutations, a lower rate of *APC* mutations and of other genes involved in the WNT pathway (such as *CTNNB1*), and an atypical mutation pattern of *BRAF*.^6^ Of note, the most commonly mutated genes in SBAs are *TP53* (38%-58%) and *KRAS* (43%-54%), followed by *APC* (18%-27%), *PIK3CA* (9%-20%), *SMAD4* (14%-17%), *CDKN2A* (3%-14%), *BRAF* (4%-11%), and *ERBB2* (7%-14%).^6-10^ Some of these molecular alterations, such as *ERBB2* mutations/amplifications and MMRd, could represent potential therapeutic targets or predictive biomarkers.^11^ However, the molecular features of SB-PCCs are unknown to date.

PCCs were initially investigated, both clinicopathologically and molecularly, in the stomach, where a signet ring cell (SRC) type has been separated from non-SRC types.^12^ The most recurrently mutated PCC-specific genes in gastric PCCs, across many studies, were *CDH1* and *RHOA*, while *CDH1* and *CTNNA1* variants cause hereditary diffuse gastric cancer.^13,14^ Interestingly, the genomically stable (GS) subtype of gastric cancer from The Cancer Genome Atlas molecular classification, which represents nearly 20% of all gastric cancers, is associated with the poorest prognosis and poor response to conventional chemotherapy, and it is enriched for “diffuse”/PCC histology, as well as for *CDH1* and *RHOA* mutations.^15,16^

The aim of our study was to identify potential driver genetic mutations underpinning SB-PCCs by next-generation sequencing (NGS), in order to gain an insight into the molecular profile of, and, it is hoped, to find possible therapeutic targets for this highly aggressive subtype.

## MATERIALS AND METHODS

### Study Population

This study was approved by the Ethics Committee of Pavia (protocol number 20140003980) and was performed in accordance with the Declaration of Helsinki. We investigated 15 surgically resected, primary nonampullary, nonhereditary SB-PCCs, all of which had already been included in previous studies of the Small Bowel Cancer Italian Consortium (accounting for 9.7% of the entire cohort of 154 SBA cases).^5,17-22^

Clinicopathologic data, including patient age at cancer diagnosis, patient sex, tumor site, predisposing immune-mediated conditions (Crohn disease or celiac disease), American Joint Committee on Cancer (AJCC) TNM stage,^23^ and lymphovascular and perineural invasion, were recorded for each case. Crohn disease was diagnosed according to the internationally agreed-on criteria, based on clinical, endoscopic, histopathologic, and radiologic features.^24^ Celiac disease was diagnosed on the basis of serum IgA antiendomysial and/or anti–tissue transglutaminase antibody positivity associated with consistent duodenal histopathologic lesions.^25^

SB-PCCs were defined as small bowel adenocarcinomas with a poorly cohesive pattern (ie, a dyshesive cell invasion pattern, characterized by a single cell or cord-like stromal invasion with or without an SRC component) representing more than 50% of the tumor, as previously described.^5^

Histologically, all SB-PCCs were reinvestigated for the presence of appendiceal-type low-grade goblet cell adenocarcinoma (GCA)–like components (ie, cohesive clusters or crypt-like structures of goblet-like cells). In addition, they were reanalyzed for the percentage of SRCs, defined as neoplastic cells featuring a cytoplasmic, globoid droplet of mucin and an eccentrically placed nucleus. SB-PCCs were divided in three histologic subtypes, including SRC-type PCCs (whose SRC component represented >90% of the neoplastic growth), combined poorly cohesive NOS and SRC carcinomas (featuring an SRC component constituting >10% but <90% of the neoplastic cellularity), and PCC-NOS (composed of <10% of SRCs), according to the criteria proposed for gastric cancer.^12^ Finally, immunohistochemistry for mismatch repair proteins was recorded, as previously described.^5^

### Molecular Analysis

The DNA was extracted from macrodissected formalin-fixed, paraffin-embedded (FFPE) slices using the QIAamp DNA FFPE Tissue Kit (Qiagen). The extracted DNA was quantified with the Qubit dsDNA HS Assay on a Qubit 3.0 fluorometer (Invitrogen) and diluted for the subsequent molecular analyses.

We used 40 ng DNA from each sample to prepare the NGS libraries with the Trusight Oncology 500 DNA kit (Illumina), which includes the analysis of single nucleotide variants (SNVs) of 523 genes and copy number variations (CNVs) of 59 genes, involved in the main cancer pathways (Supplementary Table 1; all supplemental materials can be found at *American Journal of Clinical Pathology* online), and the evaluation of MSI and tumor mutational burden (TMB).

During the protocol, the DNA was fragmented with the ultrasonicator ME220 (Covaris), amplified, and enriched for the regions of interest. Final libraries were sequenced on the NextSeq550 platform (Illumina) with a paired-end configuration (2 × 100 base pairs).

### *CDH1* Promoter Methylation Test

The methylation status of *CDH1* promoter was determined by pyrosequencing after bisulfite treatment of 100 to 300 ng tumor DNA using the EZ DNA Methylation Kit (Zymo Research). The core promoter of *CDH1* encompasses the 5ʹ upstream region and exon 1 of the gene, as annotated by the UCSC database (chr16: 68,771,035-68,772,314, GChr37/hg19 assembly). The seven CpG dinucleotides analyzed in this study fall in the 5ʹ upstream region of *CDH1* (chr16: 68,771,035-68,771,073). Bisulfite-converted DNA was amplified using TaKaRa EpiTaqTM HS reagents (Takara Bio). Primers used for polymerase chain reaction reactions were the following: forward 5ʹ-AGTAATTTTAGGTTAGAGGGTTA and reverse 5ʹ-biotin-ACCACAACCAATCAACAAC, while the sequencing primer was 5ʹ-ATTTTAGGTTAGAGGGTTAT. The cutoff value to call the presence of methylation was 10%.

### Bioinformatics and Statistics Analysis

Data were analyzed using the Illumina TSO500 Local app software version 2.0.1.4. As quality filtering criteria, we set median insert size as 75 or more and percentage of target bases with coverage greater than 100× as 75 or more. Then, we annotated Variant Call Format (vcf) files with Annovar,^26^ applying the following filters: (1) quality, 20 or higher; (2) read depth, 100× or more; (3) variant read frequency, 5% or greater; (4) exclusion of nonexonic, nonsplicing, and synonymous variants; and (5) frequency in population, 1% or less. After that, we annotated variants with Varsome, classifying them as benign, pathogenic, or of uncertain significance.^27^

We extracted only pathogenic variants and a list of genes with at least one variant. We separated samples in two ways: the first one according to Crohn and non-Crohn disease and the second one according to SRC type or non-SRC type (including PCC-NOS and combined poorly cohesive NOS and SRC carcinomas). Then, we generated oncoprints (ComplexHeatmap R package)^28^ with all the samples.

Categorical and continuous data were summarized as percentages and medians, and comparisons between groups were performed using the Fisher exact test and Mann-Whitney *U* test, respectively. A *P* value less than .05 was considered statistically significant.

## RESULTS

### Clinicopathologic and Histologic Features of SB-PCCs

The demographic and clinicopathologic data of the 15 SB-PCC cases included in the present study are summarized in **Table 1** and Supplementary Table 2. Patients were predominantly men (67%), and the median age of patients at cancer diagnosis was 52 years. Cancers arose mainly in the ileum (10 cases, 67%) and less frequently in the duodenum (3 cases, 20%) or the jejunum (2 cases, 13%). Most patients were affected by Crohn disease (12 cases, 80%); the remaining cases were associated with celiac disease (2 cases, 13%) or sporadic (1 case, 7%).

**Table 1:** Clinicopathologic Features of the 15 SB-PCCs of the Present Series

Characteristic	Value
Age at cancer diagnosis, median (25th-75th IQR), y	52 (42-59)
Female sex, No. (%)	5 (33)
Tumor site, No. (%)	
Duodenum	3 (20)
Jejunum	2 (13)
Ileum	10 (67)
Etiology, No. (%)	
Crohn disease	12 (80)
Celiac disease	2 (13)
Sporadic	1 (7)
pT stage, No. (%)	
pT3	7 (47)
pT4	8 (53)
AJCC TNM stage, No. (%)	
I	0 (0)
II	6 (40)
III	8 (53)
IV	1 (7)
Lymphovascular invasion, No. (%)	15 (100)
Perineural invasion, No. (%)	14 (93)
PCC subtypes, No. (%)	
SRC-type	3 (20)
Combined PCC-NOS and SRC carcinoma	3 (20)
PCC-NOS	9 (60)
Low-grade GCA-like component, No. (%)	3 (20)
MMRd, No. (%)	1 (7)

AJCC, American Joint Committee on Cancer (8th edition); GCA, goblet cell adenocarcinoma; IQR, interquartile range; MMRd, mismatch repair deficiency; PCC-NOS, poorly cohesive carcinoma not otherwise specified; SB-PCC, small bowel poorly cohesive carcinoma; SRC, signet ring cell.

All SB-PCCs invaded beyond the bowel muscularis propria (47% staged as pT3 and 53% as pT4). Six (40%) cancers had no evidence of metastatic disease at diagnosis (stage II), while nine (60%) cases showed lymph node and/or distant metastases at diagnosis (stages III-IV). Perineural and lymphovascular invasion were observed in 93% and 100% of SB-PCCs, respectively.

**FIGURE 1:**
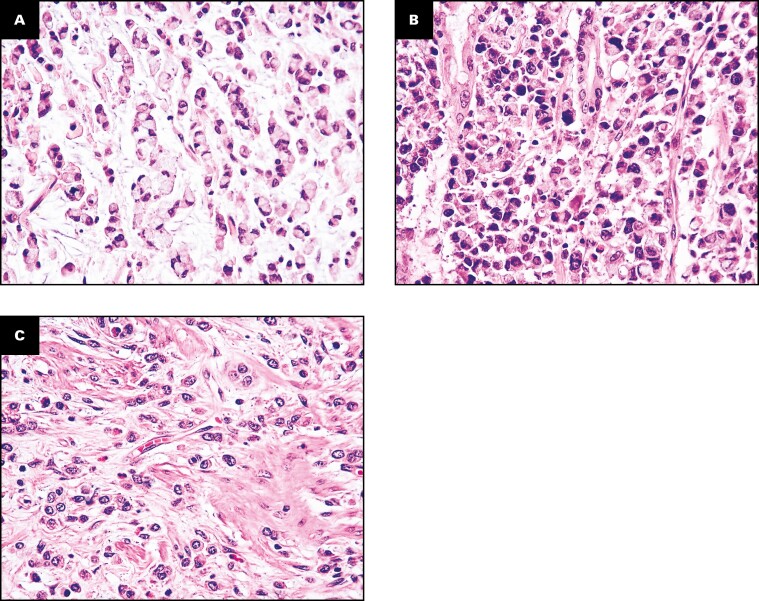
Representative images of the three histologic subtypes of small bowel poorly cohesive carcinomas (SB-PCCs). **A**, A signet ring cell (SRC)–type carcinoma, almost entirely (>90%) composed of SRCs, featuring a cytoplasmic, globoid droplet of mucin and an eccentrically placed nucleus. **B**, A combined poorly cohesive not otherwise specified and SRC carcinoma, characterized by a neoplastic growth comprising both an SRC (10%-90%) and a non-SRC poorly cohesive component. **C**, A PCC not otherwise specified (PCC-NOS), featuring a dyshesive cell invasion pattern and composed of less than 10% of SRCs. (H&E, ×400.)

Based on the percentage of SRCs, three (20%) cases were considered SRC-type SB-PCCs, three (20%) cases as “combined PCC-NOS and SRC carcinomas” (all of which with a SRC component accounting for 20%-30% of the tumor), and nine (60%) cases as PCC-NOS **Figure 1**. Three (20%) cases featured a minor (30%-40%) low-grade GCA-like component.

Only one (7%) SB-PCC (patient 12) exhibited an MMRd profile (loss of MLH1 and PMS2) and a PCC-NOS histology; this neoplasm arose in a 56-year-old male patient affected by Crohn disease; germline testing in such patient excluded Lynch syndrome.

### Molecular Findings

The analysis of the NGS results of the Trusight Oncology 500 revealed several molecular alterations in the 15 SB-PCCs. The analysis of MSI and TMB status revealed that only one sample out of 15 (patient 12) had a high MSI score (37.5) and a high TMB value (33), in keeping with its dMMR profile identified by immunohistochemistry, while the others had low MSI scores (<6) and TMB values (<10) (Supplementary Table 3). Crohn-related samples resulted in a lower TMB value median than non-Crohn-related samples (4.1 vs 9.5; *P* value of 9.63E-02 with Mann-Whitney *U* test).

The analysis of SNVs, after filtering to exclude common polymorphisms and intronic, synonymous, and bad-quality variants, revealed a total of 261 exonic/splicing variants with a range of 8 to 50 variants per sample. Most alterations were missense variants (233/261), followed by nonsense variants (11/261), frameshift deletions/insertions (8/261), splicing variants (5/261), and in-frame deletions (4/261). The Varsome tool classified the 261 variants as 44 (16.9%) pathogenic/likely pathogenic variants, 104 (39.8%) uncertain significance variants, and 113 (43.3%) benign/likely benign variants.

The 44 pathogenic variants were present in 14 of 15 tumors and were localized in 32 genes: *TP53* (8), *RHOA* (2), *ARID1A* (2), *SMAD4* (2), *MTOR* (2), *CHD4* (2), *APC* (1), *BCOR* (1), *CDH1* (1), *CTNNA1* (1), *ERBB2* (1), *ESR1* (1), *HNF1A* (1), *IDH1* (1), *KDM5C* (1), *MET* (1), *MUTYH* (1), *NKX2-1* (1), *NTRK3* (1), *PIK3CD* (1), *PPARG* (1), *PRKDC* (1), *PTCH1* (1), *RANBP2* (1), *RASA1* (1), *RECQL4* (1), *RNF43* (1), *RPS6KB1* (1), *STT3A* (1), *TAF1* (1), *TET2* (1), and *TSHR* (1). Some samples harbored two variants in the same gene: #01 in *SMAD4*, #10 in *CDH4*, and #12 in *ARID1A*. The complete list of the 44 pathogenic variants is reported in Supplementary Table 4.

The analysis of CNVs revealed that 7 of 15 samples (#03, #05, #08, #09, #11, #14, #15) harbored a total of 11 amplifications in 10 genes: *KRAS* (2), *EGFR* (1), *MYCL* (1), *CDK6* (1), *FGFR2* (1), *BRCA2* (1), *LAMP1* (1), *MYC* (1), *RICTOR* (1), and *FGF10* (1). The complete list of CNVs is reported in Supplementary Table 5.

**FIGURE 2:**
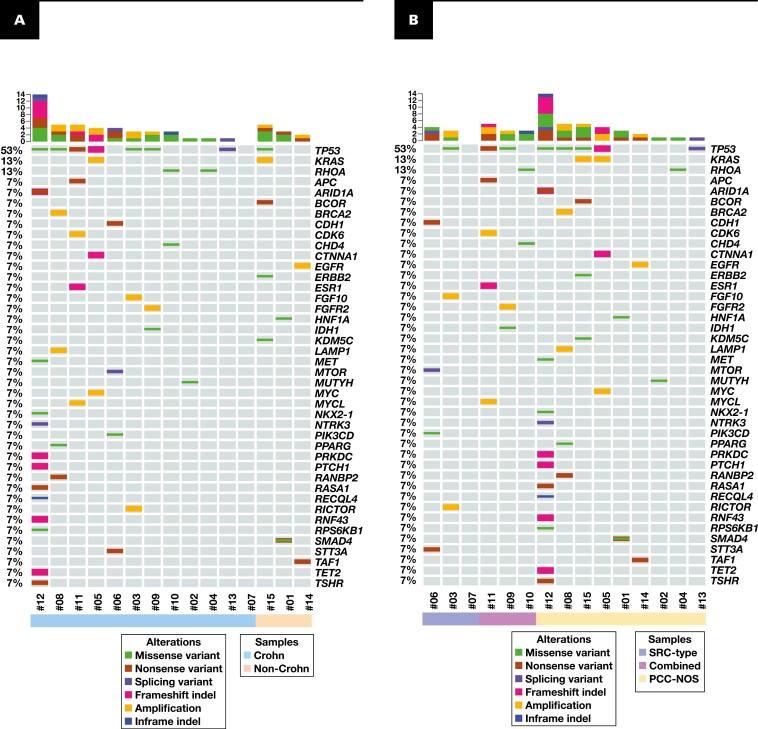
Oncoprint with pathogenic variations. Each row is a gene, sorted by the percentage of samples containing a variation on that gene. Samples are reported as columns and subdivided in groups; within each group, samples are sorted by the number of variants. Each cell highlights the presence and the type of the variants. **A**, Samples are split according to Crohn disease. **B**, Separation by poorly cohesive carcinoma (PCC) subtype (signet ring cell [SRC] type, combined SRC carcinomas and PCC not otherwise specified [PCC-NOS], and PCC-NOS).

The Oncoprint analysis of the pathogenic SNVs and CNVs was used to compare the mutational profiles in different tumor subgroups. The comparison of tumors from patients with Crohn disease (n = 12) vs those without Crohn disease (n = 3) is reported in **Figure 2A**, while the comparison of SRC-type tumors (n = 3), PCCs-NOS (n = 9), and combined PCC-NOS and SRC carcinomas (n = 3) is reported in **Figure 2B**.

*TP53* mutations were more frequently found in SB-PCCs associated with Crohn disease (58%) in comparison with cases not associated with Crohn disease (33%), although the difference did not reach statistical significance. Likewise, *TP53* mutations were more common in non-SRC-type SB-PCCs (58%), compared with those with SRC-type histology (33%). *KRAS* and *FGFR2* amplifications were identified in two (13%) cases and one (7%) case, respectively, all of which showed non-SRC-type histology. The single *IDH1*-mutated SB-PCC arose in a patient with Crohn disease and was histologically classified as a combined poorly cohesive NOS and SRC carcinoma. One SB-PCC, which was associated with celiac disease and showed a PCC-NOS histology, harbored a point mutation of the *ERBB2* gene. One Crohn-related non-SRC-type SB-PCC harbored a frameshift deletion of the *CTNN1A* gene, along with *KRAS* and *MYC* amplifications, while a *CDH1* mutation was identified in another cancer (SRC type, Crohn related). No case showed *CDH1* promoter methylation.

### Clinicopathologic Correlates of *RHOA*-Mutated SB-PCCs

**FIGURE 3:**
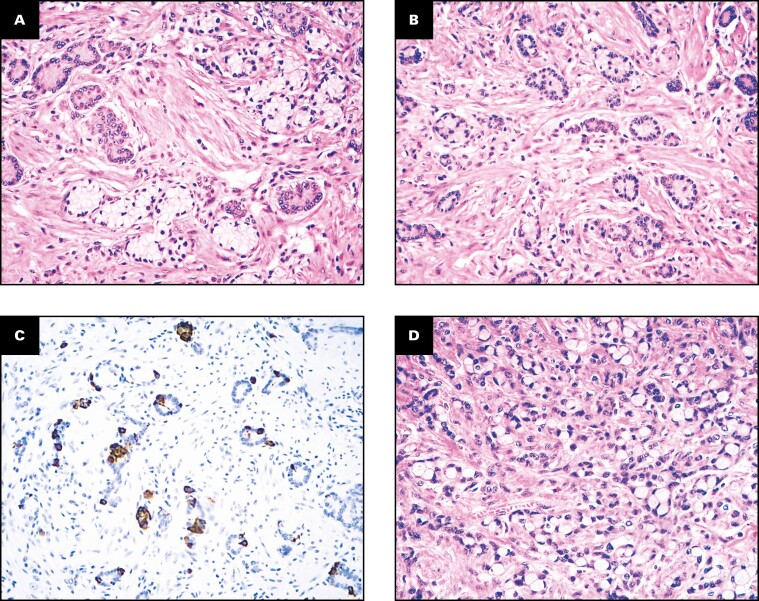
Histologic features of the ileal *RHOA*-mutated small bowel carcinoma. A superficial, minor portion of the neoplasm shows a low-grade goblet cell adenocarcinoma-like component with cohesive tumor cell clusters, including goblet-like cells with clear cytoplasm (**A**, **B**) and synaptophysin-positive neuroendocrine cells (**C**), while the deeper part of the cancer (**D**, on the lower left) features a poorly cohesive cell pattern, which accounts for more than 50% of the neoplasm. (**A**, **B**, **D**, H&E; **C**, synaptophysin immunostaining; **A**, ×200; **B**, ×100; **C**, ×100; **D**, ×400.)

Both *RHOA*-mutated SB-PCC cases arose in relatively young patients with Crohn disease. One stage II (pT4N0) MMR-proficient cancer (with the Gly17Glu mutation of the *RHOA* gene) occurred in the nonampullary duodenum of a 50-year-old man, while the other one, a stage III (pT3N1) MMR-proficient SBA (with the Tyr42Cys mutation), was found in the ileum of a 52-year-old female patient. Histologically, we observed that both *RHOA*-mutated SB-PCCs featured a minor (30%-40%) and superficial, appendiceal-type, low-grade GCA-like component **Figure 3**, whereas only one (8%) of the remaining 13 *RHOA* wild-type (WT) SB-PCCs showed a focal GCA-like component (*P* = .029). In both cases, the appendix was without evidence of disease. The PCC component was classified as PCC-NOS in the duodenal case and as combined PCC-NOS and SRCC in the ileal *RHOA*-mutated carcinoma. Both *RHOA*-mutated cases were *TP53*-WT.

## DISCUSSION

In the present study, we have gained insights into the molecular landscape of SB-PCCs, thus contributing to the characterization of this histologic subtype of nonampullary SBAs with distinct clinicopathologic (including frequent association with Crohn disease) and prognostic features in comparison with conventional SBAs.^5^ A different SB-PCC molecular profile, compared with the SBAs in general and with conventional-type or SRC CRCs, emerged, particularly for (1) the lack of *KRAS* and *PIK3CA* point mutations, (2) the rarity of the MMR deficiency/MSI profile, and (3) the presence of the *RHOA* mutation, typical of PCCs of the stomach.

We identified *TP53* as the most frequently altered gene in SB-PCCs (53%), with mutation rates similar to those reported by previous studies analyzing SBAs (38%-58%),^2,6-8^ or gastric carcinomas (57% in intestinal-type cancers and 41% in diffuse-type carcinomas).^14^ The higher frequency of *TP53* mutation in SB-PCCs associated with Crohn disease found in our series confirmed previous findings on Crohn disease–associated SBAs in general, which have been reported to show *TP53* mutations in about 70% of cases.^8,29^ Interestingly, *TP53* mutations were more common in non-SRC-type SB-PCCs (58%) compared with SRC-type histology (33%), with a figure similar to that observed in diffuse-type gastric carcinomas by Kwon et al.^13^

One of the more novel and intriguing findings of our investigation was the identification of *RHOA* gene mutations in two (13%) SB-PCCs (one with Tyr42Cys and the other one with Gly17Glu mutation). *RHOA* is a gene encoding for a member of the Rho family, a group of small GTPases with structural and signaling similarity to Ras proteins involved in the regulation of cytoskeletal dynamics, cell migration, and cell cycle, which has recently been identified as a promising therapeutic target in gastric cancer.^30,31^*RHOA* mutations have been described in gastric cancers, where they have been shown to be almost exclusively present in diffuse-type gastric carcinomas (14%-25%)^13,32,33^ with the exception of a few intestinal-type/mixed-type gastric cancers (with anastomosing glands),^34^ in appendiceal GCA,^35^ and in some acute leukemias.^36^ On the contrary, they seem to be extremely rare in colorectal adenocarcinoma (0.7%), as well as in other solid tumors.^36^ To the best of our knowledge, *RHOA* mutations have not been reported in SBAs. However, *RHOA* is not commonly included in limited targeted sequencing panels, and thus the true occurrence of *RHOA* mutations in SBAs has not been thoroughly investigated. The most frequently reported *RHOA* mutation in diffuse gastric cancer, *RHOA* Tyr42Cys, found also in one of the two *RHOA*-mutated SB-PCCs of our series, seems to behave as a gain-of-function mutation, enhancing cancer cell motility and survival and inducing an increased ability to bind the effector protein Rock, with a subsequent increase in stress-fiber formation.^31,37^ Both *RHOA*-mutated SB-PCCs were Crohn disease–associated *TP53*-WT and featured non-SRC histology, similar to observations in diffuse-type gastric cancers.^13^ In addition, both cases showed superficial “appendiceal-type, low-grade, GCA”-like foci with focal positivity for neuroendocrine markers. The latter histologic feature is intriguing because (1) *RHOA* mutations have also been described in appendiceal GCAs,^35^ and (2) *RHOA*-mutated diffuse-type gastric cancers have been reported to be characterized by a superficial SRC or tubular differentiation and a deep invasive PCC-NOS, more desmoplastic, component.^13,38^ Whether such *RHOA*-mutated small bowel cancers with minor, low-grade GCA-like components are better classified as “extra-appendiceal GCAs with prominent PCC component” rather than as SB-PCCs requires further evidence. Diffuse-type and, in particular, the GS molecular subtype of gastric cancer are also enriched for *CLDN18*-*ARHGAP* fusions, which seem to be mutually exclusive with *RHOA* and *CDH1* mutations. However, we did not include *CLDN18-ARHGAP* analysis in the present investigation on SB-PCCs, also considering that data concerning the fusion mainly stem from retrospective studies using different detection methods.^39,40^

*KRAS* mutations are frequently detected in SBAs in general (44%-54%^6-8^) and in SRC carcinomas of the colon-rectum (50%),^41^ whereas they appear to be never or very rarely detected in SB-PCCs (with no case of our series harboring *KRAS* point mutations). In addition, *KRAS* mutations have been reported in 24% to 50% of inflammatory bowel disease (IBD)–associated SBAs,^6,8,29^ suggesting that the lack of *KRAS* point mutations is more likely related to the SB-PCC histology than to IBD-related etiology. Similarly, *KRAS* mutations are rare in intestinal-type and diffuse/poorly cohesive gastric carcinomas (<10% of cases),^14^ appendiceal GCAs (0%-6%), and SRC adenocarcinomas (0%).^35,42-44^ Interestingly, Jun et al^45^ found no *KRAS* mutations in four SRC SBAs. Two (13%) cases, one of which was associated with celiac disease and the other one with Crohn disease, harbored *KRAS* amplifications. This result is intriguing if we consider a recent study reporting a strong relationship between a history of IBD and the occurrence of *KRAS*-amplified metastatic CRCs.^46^*KRAS* amplifications have been reported in about 19% of SBAs by Hänninen et al.^7^ Several studies demonstrated in different cancer entities that amplifications occurred mutually exclusively with mutations in *KRAS* and that amplifications were usually associated with poor prognosis in most cases.^47-49^

Recent molecular studies identified *APC*, a Wnt-pathway gene whose mutations represent one of the early events in the colorectal carcinogenic process, as one of the most commonly mutated genes in SBAs (13%-27%),^2,6-8^ although its alterations occurred more rarely (0%-6%) in Crohn disease–associated SBAs.^6,8,29^ In keeping with these observations, *APC* pathogenic mutation was identified only in one (7%) case of our series, which was enriched with Crohn disease–related cases. In addition, a pathogenic mutation, likely somatic, in *CTNNA1* (another WNT pathway–related gene that encodes for α1 catenin, a protein involved in cell adhesion and whose mutations are found in a minority of hereditary diffuse gastric cancers^50^) was identified in a Crohn disease–associated, *APC-*WT PCC-NOS, whereas no mutation involving the *CTNNB1* gene encoding β-catenin was found in our series. Likewise, *PIK3CA*, which has been identified by various authors as one of the most frequently mutated genes in SBAs (8%-33%),^6-8,29^ with a trend for more frequent *PIK3CA* alterations in Crohn disease–associated SBAs (up to 43%),^8^ was never found to be mutated in SB-PCCs of our series.

Surprisingly, although the diffuse/discohesive growth pattern represents the main histologic distinctive feature of SB-PCCs, only one SB-PCC of our series showed a pathogenic mutation of the *CDH1* gene, which encodes for E-cadherin, a calcium-dependent transmembrane protein mediating cellular adhesion, whose loss of function causes adhesion and signaling impairment and increased cell survival and migration. No SB-PCC case harboring *CDH1* promoter hypermethylation, another possible mechanism of *CDH1* inactivation, was identified. Aparicio et al^8^ identified *CDH1* mutations in only 0.8% of their SBAs. Germline *CDH1* mutations are associated with hereditary diffuse gastric cancer,^14^ while sporadic mutations are relatively common (about 20%) in poorly cohesive gastric carcinomas^13,14^ and in appendiceal GCAs, where these mutations could represent additional hits transforming the neoplasm into a poorly differentiated adenocarcinoma with a dyshesive cell spread.^43^

In our series, one combined PCC-NOS and SRC carcinoma (with an SRC component accounting for 30% of the neoplastic growth) arising in a 39-year-old woman affected by Crohn disease showed an *IDH1* pathogenic mutation. *IDH1* mutations, which are common in gliomas, acute myeloid leukemia, intrahepatic cholangiocarcinomas, sinonasal undifferentiated carcinomas, enchondromas, and chondrosarcomas, have also been identified in intestinal cancers.^51-54^ Hartman et al^52^ found that *IDH1*-mutated intestinal cancers were more common in IBD-related adenocarcinomas in comparison to sporadic cases and were associated with a low-grade tubuloglandular histology. The association of *IDH1* molecular alterations with IBD-related neoplasms appeared to be confirmed in the small intestine by both Aparicio et al,^8^ who identified *IDH1* mutations in 1% of their sporadic SBAs in contrast to 28.6% of Crohn disease–related SBAs, and by Liao et al,^29^ who observed *IDH1* mutation exclusively in Crohn disease–related SBAs (18%). Our findings, in combination with those of Liao et al,^29^ who described one case of *IDH1*-mutated SBA showing an SRC component, expand the possible histologic spectrum of *IDH1*-mutated SBAs. The identification of *IDH1* mutations in cancers such as SB-PCCs, whose prognosis is dismal to this day and whose therapeutic options still remain limited, might be of potential clinical relevance in the near future, as *IDH1* mutations represent well-established therapeutic targets in other cancer types.^55^ In addition, we found one SB-PCC associated with celiac disease with a point mutation of the *ERBB2* gene, which has been reported to show promising targetable alterations (mutations or amplifications) in up to 23% of SBAs.^11^ A Crohn-related SB-PCC harbored an *FGFR2* amplification, which has already been reported to be more common in intestinal cancers associated with Crohn disease^51^ and in poorly cohesive gastric cancers, where it seems to be associated with increasing sensitivity to FRFR2 inhibitors.^56^ Finally, our molecular data confirm our previously described immunohistochemical findings on the rarity of MMRd/MSI in SB-PCCs.^5^ The only MSI SB-PCC was a PCC-NOS occurring in a 56-year-old male patient affected by Crohn disease, which showed no mutations in the MMR genes and had a higher TMB (32.8 mutations/Mb) compared with the other SB-PCCs. Despite the high TMB of this MMRd/MSI SB-PCC, we have found that Crohn disease–associated SB-PCCs harbored a significantly lower TMB compared with cases not related to Crohn disease.

It should be added that Yaeger et al^51^ found higher rates of *KRAS* (33%) and *APC* (39%) mutations in intestinal, mainly colorectal, carcinomas associated with Crohn disease in comparison with SB-PCCs related to Crohn disease of our study (0% and 8%, respectively). On the other hand, no *RHOA* mutation was identified in a whole-exome sequencing analysis of 32 IBD-associated colorectal cancers, as opposed to our SB-PCCs related to Crohn disease, which showed *RHOA* mutations in 17% of cases.^57^ Nevertheless, in the study by Robles et al,^57^ genes involved in the Rho and Rac GTPase network were reported to be recurrently mutated in 30% of cases.

The main limitation of this study is the relatively small sample size of the analyzed case series. However, SB-PCCs represent a very uncommon form of tumor, and in our study, we performed, for the first time, a wide molecular characterization that has highlighted several interesting results that deserve to be further investigated. The similarity of molecular patterns between gastric PCCs and presently investigated SB-PCCs is interesting and may suggest a histotype-dependent pattern. Given the high frequency of Crohn disease–associated cases in our cohort, it remains to be further investigated whether a few molecular findings, such as *RHOA* mutations, are specifically related to PCC histology or whether they are secondary to the molecular pathogenesis of Crohn-related SBAs. However, at least some molecular findings found in our SB-PCC cases, such as the lack of *KRAS* and *PIK3CA* point mutations, are likely histotype dependent, as they are observed in SB-PCCs with or without Crohn disease and are not a feature of Crohn disease–associated SBAs in general.^6,8,29^

In conclusion, we found the occurrence of *RHOA* mutations, which are reminiscent of the diffuse subtype of gastric cancers or appendiceal GCAs, in 13% of SB-PCC cases, while mutations commonly involved in colorectal and small bowel adenocarcinomas (such as those involving *KRAS*, *PIK3CA*) are rarely, if ever, seen in SB-PCCs. In addition, we identified, albeit rarely, potentially targetable alterations, such as *IDH1* or *ERBB2* mutations or MMRd/MSI, in such aggressive cancers, most of which (80%) were associated with Crohn disease.

## Supplementary Material

aqac161_suppl_Supplementary_Tables
