# Supplementary material for: Molecular Landscape and Association With Crohn Disease of Poorly Cohesive Carcinomas of the Nonampullary Small Bowel
Source: Am J Clin Pathol. 2023 Feb 22;159(4):315–24. doi: 10.1093/ajcp/aqac161 (PMC10071142; doi:10.1093/ajcp/aqac161)
Supplement: aqac161_suppl_Supplementary_Tables [file aqac161_suppl_supplementary_tables.docx]

**Supplementary Materials**

**Supplementary Table 1: List of the 523 genes (the 59 genes in bold are analyzed also for CNV detection).**

| **Genes** | | | | | | | | | | | |
| --- | --- | --- | --- | --- | --- | --- | --- | --- | --- | --- | --- |
| *ABL1* | *BMPR1A* | *CSF1R* | *ERCC5* | *FLI1* | *HIST1H3I* | *KDR* | *MRE11A* | *PAX3* | *PTCH1* | *SDHD* | *TCF7L2* |
| *ABL2* | ***BRAF*** | *CSF3R* | *ERG* | *FLT1* | *HIST1H3J* | *KEAP1* | *MSH2* | *PAX5* | ***PTEN*** | *SETBP1* | *TERC* |
| *ACVR1* | ***BRCA1*** | *CSNK1A1* | *ERRFI1* | *FLT3* | *HIST2H3A* | *KEL* | *MSH3* | *PAX7* | *PTPN11* | *SETD2* | *TERT* |
| *ACVR1B* | ***BRCA2*** | *CTCF* | ***ESR1*** | *FLT4* | *HIST2H3C* | *KIF5B* | *MSH6* | *PAX8* | *PTPRD* | *SF3B1* | *TET1* |
| *AKT1* | *BRD4* | *CTLA4* | *ETS1* | *FOXA1* | *HIST2H3D* | ***KIT*** | *MST1* | *PBRM1* | *PTPRS* | *SH2B3* | *TET2* |
| ***AKT2*** | *BRIP1* | *CTNNA1* | *ETV1* | *FOXL2* | *HIST3H3* | *KLF4* | *MST1R* | *PDCD1* | *PTPRT* | *SH2D1A* | *TFE3* |
| *AKT3* | *BTG1* | *CTNNB1* | *ETV4* | *FOXO1* | *HLA-A* | *KLHL6* | *MTOR* | *PDCD1LG2* | *QKI* | *SHQ1* | ***TFRC*** |
| ***ALK*** | *BTK* | *CUL3* | *ETV5* | *FOXP1* | *HLA-B* | *KMT2B* | *MUTYH* | ***PDGFRA*** | *RAB35* | *SLIT2* | *TGFBR1* |
| *ALOX12B* | *C11orf30* | *CUX1* | *ETV6* | *FRS2* | *HLA-C* | *KMT2C* | *MYB* | ***PDGFRB*** | *RAC1* | *SLX4* | *TGFBR2* |
| *ANKRD11* | *CALR* | *CXCR4* | *EWSR1* | *FUBP1* | *HNF1A* | *KMT2D* | ***MYC*** | *PDK1* | *RAD21* | *SMAD2* | *TMEM127* |
| *ANKRD26* | *CARD11* | *CYLD* | *EZH2* | *FYN* | *HNRNPK* | ***KRAS*** | ***MYCL1*** | *PDPK1* | *RAD50* | *SMAD3* | *TMPRSS2* |
| *APC* | *CASP8* | *DAXX* | *FAM123B* | *GABRA6* | *HOXB13* | ***LAMP1*** | ***MYCN*** | *PGR* | *RAD51* | *SMAD4* | *TNFAIP3* |
| ***AR*** | *CBFB* | *DCUN1D1* | *FAM175A* | *GATA1* | *HRAS* | *LATS1* | *MYD88* | *PHF6* | *RAD51B* | *SMARCA4* | *TNFRSF14* |
| *ARAF* | *CBL* | *DDR2* | *FAM46C* | *GATA2* | *HSD3B1* | *LATS2* | *MYOD1* | *PHOX2B* | *RAD51C* | *SMARCB1* | *TOP1* |
| *ARFRP1* | ***CCND1*** | *DDX41* | *FANCA* | *GATA3* | *HSP90AA1* | *LMO1* | *NAB2* | *PIK3C2B* | *RAD51D* | *SMARCD1* | *TOP2A* |
| *ARID1A* | *CCND2* | *DHX15* | *FANCC* | *GATA4* | *ICOSLG* | *LRP1B* | *NBN* | *PIK3C2G* | *RAD52* | *SMC1A* | *TP53* |
| *ARID1B* | ***CCND3*** | *DICER1* | *FANCD2* | *GATA6* | *ID3* | *LYN* | *NCOA3* | *PIK3C3* | *RAD54L* | *SMC3* | *TP63* |
| *ARID2* | ***CCNE1*** | *DIS3* | *FANCE* | *GEN1* | *IDH1* | *LZTR1* | *NCOR1* | ***PIK3CA*** | ***RAF1*** | *SMO* | *TRAF2* |
| *ARID5B* | *CD274* | *DNAJB1* | *FANCF* | *GID4* | *IDH2* | *MAGI2* | *NEGR1* | ***PIK3CB*** | *RANBP2* | *SNCAIP* | *TRAF7* |
| *ASXL1* | *CD276* | *DNMT1* | *FANCG* | *GLI1* | *IFNGR1* | *MALT1* | *NF1* | *PIK3CD* | *RARA* | *SOCS1* | *TSC1* |
| *ASXL2* | *CD74* | *DNMT3A* | *FANCI* | *GNA11* | *IGF1* | *MAP2K1* | *NF2* | *PIK3CG* | *RASA1* | *SOX10* | *TSC2* |
| ***ATM*** | *CD79A* | *DNMT3B* | *FANCL* | *GNA13* | *IGF1R* | *MAP2K2* | *NFE2L2* | *PIK3R1* | *RB1* | *SOX17* | *TSHR* |
| *ATR* | *CD79B* | *DOT1L* | *FAS* | *GNAQ* | *IGF2* | *MAP2K4* | *NFKBIA* | *PIK3R2* | *RBM10* | *SOX2* | *U2AF1* |
| *ATRX* | *CDC73* | *E2F3* | *FAT1* | *GNAS* | *IKBKE* | *MAP3K1* | *NKX2-1* | *PIK3R3* | *RECQL4* | *SOX9* | *VEGFA* |
| *AURKA* | *CDH1* | *EED* | *FBXW7* | *GPR124* | *IKZF1* | *MAP3K13* | *NKX3-1* | *PIM1* | *REL* | *SPEN* | *VHL* |
| *AURKB* | *CDK12* | *EGFL7* | ***FGF1*** | *GPS2* | *IL10* | *MAP3K14* | *NOTCH1* | *PLCG2* | ***RET*** | *SPOP* | *VTCN1* |
| *AXIN1* | ***CDK4*** | ***EGFR*** | ***FGF10*** | *GREM1* | *IL7R* | *MAP3K4* | *NOTCH2* | *PLK2* | *RFWD2* | *SPTA1* | *WISP3* |
| *AXIN2* | ***CDK6*** | *EIF1AX* | ***FGF14*** | *GRIN2A* | *INHA* | *MAPK1* | *NOTCH3* | *PMAIP1* | *RHEB* | *SRC* | *WT1* |
| *AXL* | *CDK8* | *EIF4A2* | ***FGF19*** | *GRM3* | *INHBA* | *MAPK3* | *NOTCH4* | *PMS1* | *RHOA* | *SRSF2* | *XIAP* |
| *B2M* | *CDKN1A* | *EIF4E* | ***FGF2*** | *GSK3B* | *INPP4A* | *MAX* | *NPM1* | *PMS2* | ***RICTOR*** | *STAG1* | *XPO1* |
| *BAP1* | *CDKN1B* | *EML4* | ***FGF23*** | *H3F3A* | *INPP4B* | *MCL1* | ***NRAS*** | *PNRC1* | *RIT1* | *STAG2* | *XRCC2* |
| *BARD1* | *CDKN2A* | *EP300* | ***FGF3*** | *H3F3B* | *INSR* | *MDC1* | ***NRG1*** | *POLD1* | *RNF43* | *STAT3* | *YAP1* |
| *BBC3* | *CDKN2B* | *EPCAM* | ***FGF4*** | *H3F3C* | *IRF2* | ***MDM2*** | *NSD1* | *POLE* | *ROS1* | *STAT4* | *YES1* |
| *BCL10* | *CDKN2C* | *EPHA3* | ***FGF5*** | *HGF* | *IRF4* | ***MDM4*** | *NTRK1* | *PPARG* | *RPS6KA4* | *STAT5A* | *ZBTB2* |
| *BCL2* | *CEBPA* | *EPHA5* | ***FGF6*** | *HIST1H1C* | *IRS1* | *MED12* | *NTRK2* | *PPM1D* | ***RPS6KB1*** | *STAT5B* | *ZBTB7A* |
| *BCL2L1* | *CENPA* | *EPHA7* | ***FGF7*** | *HIST1H2BD* | *IRS2* | *MEF2B* | *NTRK3* | *PPP2R1A* | *RPS6KB2* | *STK11* | *ZFHX3* |
| *BCL2L11* | *CHD2* | *EPHB1* | ***FGF8*** | *HIST1H3A* | *JAK1* | *MEN1* | *NUP93* | *PPP2R2A* | *RPTOR* | *STK40* | *ZNF217* |
| *BCL2L2* | *CHD4* | ***ERBB2*** | ***FGF9*** | *HIST1H3B* | ***JAK2*** | ***MET*** | *NUTM1* | *PPP6C* | *RUNX1* | *SUFU* | *ZNF703* |
| *BCL6* | ***CHEK1*** | ***ERBB3*** | ***FGFR1*** | *HIST1H3C* | *JAK3* | *MGA* | *PAK1* | *PRDM1* | *RUNX1T1* | *SUZ12* | *ZRSR2* |
| *BCOR* | ***CHEK2*** | *ERBB4* | ***FGFR2*** | *HIST1H3D* | *JUN* | *MITF* | *PAK3* | *PREX2* | *RYBP* | *SYK* |  |
| *BCORL1* | *CIC* | ***ERCC1*** | ***FGFR3*** | *HIST1H3E* | *KAT6A* | *MLH1* | *PAK7* | *PRKAR1A* | *SDHA* | *TAF1* |  |
| *BCR* | *CREBBP* | ***ERCC2*** | ***FGFR4*** | *HIST1H3F* | *KDM5A* | *MLL* | *PALB2* | *PRKCI* | *SDHAF2* | *TBX3* |  |
| *BIRC3* | *CRKL* | *ERCC3* | *FH* | *HIST1H3G* | *KDM5C* | *MLLT3* | *PARK2* | *PRKDC* | *SDHB* | *TCEB1* |  |
| *BLM* | *CRLF2* | *ERCC4* | *FLCN* | *HIST1H3H* | *KDM6A* | *MPL* | *PARP1* | *PRSS8* | *SDHC* | *TCF3* |  |

**Supplementary Table 2: List of the 15 small bowel cancer patients and clinico-pathological characteristics**

| **Sample ID** | **Gender** | **Age at cancer diagnosis** | **Etiology** | **PCC subtype** | **Tumor site** | **pT stage** | **TNM stage** | **Lymphovascular invasion** | **Perineural invasion** | **GCA-like component** | **MMRd** |
| --- | --- | --- | --- | --- | --- | --- | --- | --- | --- | --- | --- |
| #01 | M | 52 | sporadic | PCC-NOS | jejunum | T4 | III | yes | yes | no | no |
| #02 | F | 59 | Crohn disease | PCC-NOS | ileum | T3 | II | yes | no | no | no |
| #03 | M | 35 | Crohn disease | SRC-type | ileum | T3 | II | yes | yes | no | no |
| #04 | M | 50 | Crohn disease | PCC-NOS | duodenum | T4 | II | yes | yes | yes | no |
| #05 | M | 54 | Crohn disease | PCC-NOS | ileum | T4 | III | yes | yes | no | no |
| #06 | M | 69 | Crohn disease | SRC-type | ileum | T4 | III | yes | yes | no | no |
| #07 | M | 75 | Crohn disease | SRC-type | ileum | T3 | II | yes | yes | yes | no |
| #08 | F | 31 | Crohn disease | PCC-NOS | jejunum | T3 | II | yes | yes | no | no |
| #09 | F | 39 | Crohn disease | combined PCC-NOS and SRC carcinoma | ileum | T4 | II | yes | yes | no | no |
| #10 | F | 52 | Crohn disease | combined PCC-NOS and SRC carcinoma | ileum | T3 | III | yes | yes | yes | no |
| #11 | M | 52 | Crohn disease | combined PCC-NOS and SRC carcinoma | ileum | T4 | III | yes | yes | no | no |
| #12 | M | 56 | Crohn disease | PCC-NOS | ileum | T3 | III | yes | yes | no | yes |
| #13 | M | 43 | Crohn disease | PCC-NOS | ileum | T4 | III | yes | yes | no | no |
| #14 | M | 72 | celiac disease | PCC-NOS | duodenum | T3 | IV | yes | yes | no | no |
| #15 | F | 42 | celiac disease | PCC-NOS | duodenum | T4 | III | yes | yes | no | no |

Legend: AJCC: American Joint Committee on Cancer (8th edition); F: female; GCA: goblet cell adenocarcinoma; M: male; MMR-d: mismatch repair deficiency; PCC-NOS: poorly cohesive carcinoma not otherwise specified; SB-PCC: small bowel poorly cohesive carcinoma; SD: standard deviation; SRC: signet ring cell

**Supplementary Table 3: Tumor mutational burden (TMB) and microsatellite instability (MSI) of the 15 samples.**

| **Sample ID** | **TMB*** | **MSI**** |
| --- | --- | --- |
| #01 | 9,5 | 3,7 |
| #02 | 0 | 1,45 |
| #03 | 2,4 | 2,97 |
| #04 | 4,8 | 2,27 |
| #05 | 2,4 | 1,08 |
| #06 | 8,9 | 3,03 |
| #07 | 2,5 | 1,01 |
| #08 | 9 | 3,51 |
| #09 | 1,6 | 2,13 |
| #10 | 3,3 | 5,88 |
| #11 | 8,7 | 0 |
| #12 | 32,8 | 37,5 |
| #13 | 5 | 0 |
| #14 | 9,5 | 3,33 |
| #15 | 8,1 | 0 |
|  |  |  |
| * number of mutations/Megabase | | |
| ** % of unstable microsatellites | | |

**Supplementary Table 4: Pathogenic SNVs**

| **Sample ID** | **Chr** | **Position** | **Gene** | **Transcript** | **cDNA** | **Protein** | **Variant type** | **VAF** |
| --- | --- | --- | --- | --- | --- | --- | --- | --- |
| #01 | 12 | 121431404 | *HNF1A* | NM_000545.6 | c.608G>A | p.(Arg203His) | missense variant | 0.13 |
| #01 | 18 | 48591891 | *SMAD4* | NM_005359.5 | c.1054G>T | p.(Gly352Ter) | nonsense variant | 0.48 |
| #01 | 18 | 48591928 | *SMAD4* | NM_005359.5 | c.1091T>G | p.(Leu364Trp) | missense variant | 0.09 |
| #02 | 1 | 45798117 | *MUTYH* | NM_001128425.1 | c.734G>A | p.(Arg245His) | missense variant | 0.44 |
| #03 | 17 | 7578191 | *TP53* | NM_000546.5 | c.658T>C | p.(Tyr220His) | missense variant | 0.28 |
| #04 | 3 | 49412973 | *RHOA* | NM_001664.3 | c.50G>A | p.(Gly17Glu) | missense variant | 0.09 |
| #05 | 5 | 138163304 | *CTNNA1* | NM_001903.4 | c.960_963delCGAC | p.(Asp321ArgfsTer47) | frameshift deletion | 0.10 |
| #05 | 17 | 7577569 | *TP53* | NM_000546.5 | c.705_712dupCTACATGT | p.(Cys238SerfsTer12) | frameshift insertion | 0.15 |
| #06 | 1 | 9782408 | *PIK3CD* | NM_005026.3 | c.2341G>A | p.(Gly781Arg) | missense variant | 0.06 |
| #06 | 1 | 11186678 | *MTOR* | NM_004958.3 | c.6526+1G>A | - | splicing variant | 0.06 |
| #06 | 11 | 125490685 | *STT3A* | NM_152713.4 | c.2098C>T | p.(Arg700Ter) | nonsense variant | 0.05 |
| #06 | 16 | 68857382 | *CDH1* | NM_004360.4 | c.2017C>T | p.(Gln673Ter) | nonsense variant | 0.06 |
| #08 | 2 | 109382853 | *RANBP2* | NM_006267.4 | c.5858C>A | p.(Ser1953Ter) | nonsense variant | 0.14 |
| #08 | 3 | 12434173 | *PPARG* | NM_015869.4 | c.541C>T | p.(Arg181Trp) | missense variant | 0.49 |
| #08 | 17 | 7577538 | *TP53* | NM_000546.5 | c.743G>A | p.(Arg248Gln) | missense variant | 0.22 |
| #09 | 2 | 209113113 | *IDH1* | NM_005896.3 | c.394C>T | p.(Arg132Cys) | missense variant | 0.09 |
| #09 | 17 | 7578541 | *TP53* | NM_000546.5 | c.389T>C | p.(Leu130Pro) | missense variant | 0.28 |
| #10 | 3 | 49412898 | *RHOA* | NM_001664.3 | c.125A>G | p.(Tyr42Cys) | missense variant | 0.18 |
| #10 | 12 | 6696991 | *CHD4* | NM_001273.3 | c.3576_3590delGATGCTGACGCATCT | p.(Met1192_Leu1197delinsIle) | inframe deletion | 0.07 |
| #10 | 12 | 6697010 | *CHD4* | NM_001273.3 | c.3571A>C | p.(Lys1191Gln) | missense variant | 0.07 |
| #11 | 5 | 112175639 | *APC* | NM_000038.5 | c.4348C>T | p.(Arg1450Ter) | nonsense variant | 0.13 |
| #11 | 6 | 152201810 | *ESR1* | NM_000125.3 | c.665delC | p.(Pro222GlnfsTer29) | frameshift deletion | 0.06 |
| #11 | 17 | 7579503 | *TP53* | NM_000546.5 | c.184G>T | p.(Glu62Ter) | nonsense variant | 0.18 |
| #12 | 1 | 11298673 | *MTOR* | NM_004958.3 | c.1788C>T | c.1788C>T(p.(Gly596=)) | synonymous variant (splicing) | 0.15 |
| #12 | 1 | 27057850 | *ARID1A* | NM_006015.4 | c.1558C>T | p.(Gln520Ter) | nonsense variant | 0.15 |
| #12 | 1 | 27097622 | *ARID1A* | NM_006015.4 | c.3216delA | p.(Lys1072AsnfsTer21) | frameshift deletion | 0.17 |
| #12 | 4 | 106193850 | *TET2* | NM_001127208.2 | c.4317dupA | p.(Arg1440ThrfsTer38) | frameshift insertion | 0.13 |
| #12 | 5 | 86628434 | *RASA1* | NM_002890.2 | c.803T>G | p.(Leu268Ter) | nonsense variant | 0.16 |
| #12 | 7 | 116417460 | *MET* | NM_001127500.2 | c.3331T>C | p.(Tyr1111His) | missense variant | 0.44 |
| #12 | 8 | 48746799 | *PRKDC* | NM_006904.6 | c.8109delA | p.(Arg2704AlafsTer?) | frameshift deletion | 0.12 |
| #12 | 8 | 145737107 | *RECQL4* | NM_004260.3 | c.3458_3460delGAG | p.(Glu1153del) | inframe deletion | 0.06 |
| #12 | 9 | 98211549 | *PTCH1* | NM_000264.3 | c.3606delC | p.(Ser1203AlafsTer52) | frameshift deletion | 0.08 |
| #12 | 14 | 36987088 | *NKX2-1* | NM_001079668.2 | c.601G>A | p.(Ala201Thr) | missense variant | 0.22 |
| #12 | 14 | 81422187 | *TSHR* | NM_000369.2 | c.163C>T | p.(Gln55Ter) | nonsense variant | 0.11 |
| #12 | 15 | 88679129 | *NTRK3* | NM_001012338.2 | c.907+1G>C | - | splicing variant | 0.09 |
| #12 | 17 | 7578271 | *TP53* | NM_000546.5 | c.578A>G | p.(His193Arg) | missense variant | 0.17 |
| #12 | 17 | 56448298 | *RNF43* | NM_017763.5 | c.349delC | p.(Arg117AlafsTer41) | frameshift deletion | 0.13 |
| #12 | 17 | 58013826 | *RPS6KB1* | NM_003161.3 | c.1043C>T | p.(Ala348Val) | missense variant (splicing) | 0.17 |
| #13 | 17 | 7578556 | *TP53* | NM_000546.5 | c.376-2A>G | - | splicing variant | 0.16 |
| #14 | 23 | 70618465 | *TAF1* | NM_001286074.1 | c.3724C>T | p.(Arg1242Ter) | nonsense variant | 0.06 |
| #15 | 17 | 7577538 | *TP53* | NM_000546.5 | c.743G>A | p.(Arg248Gln) | missense variant | 0.20 |
| #15 | 17 | 37881332 | *ERBB2* | NM_004448.3 | c.2524G>A | p.(Val842Ile) | missense variant | 0.17 |
| #15 | 23 | 39922899 | *BCOR* | NM_001123385.1 | c.3809G>A | p.(Trp1270Ter) | nonsense variant | 0.14 |
| #15 | 23 | 53239931 | *KDM5C* | NM_004187.3 | c.1510G>A | p.(Val504Met) | missense variant | 0.13 |

**Supplementary Table 5: CNVs**

| **Sample ID** | **Gene** | **Fold Change** |
| --- | --- | --- |
| #03 | *RICTOR* | 1.693 |
| #03 | *FGF10* | 1.485 |
| #05 | *MYC* | 1.473 |
| #05 | *KRAS* | 3.077 |
| #08 | *BRCA2* | 1.696 |
| #08 | *LAMP1* | 1.588 |
| #09 | *FGFR2* | 1.466 |
| #11 | *MYCL* | 1.467 |
| #11 | *CDK6* | 1.400 |
| #14 | *EGFR* | 1.676 |
| #15 | *KRAS* | 5.503 |
